# 646. Bridging the Gap: Using Standardized Videos for Wound Care Education

**DOI:** 10.1093/jbcr/irag033.483

**Published:** 2026-04-06

**Authors:** Lauren Baxley, Miranda L Yelvington, Morgan Colson, Christine M Grauer

**Affiliations:** Arkansas Children's Burn Program, Little Rock, AR; Arkansas Children's Burn Program, Little Rock, AR; Arkansas Children's Burn Program, Little Rock, AR; Arkansas Children's Burn Program, Little Rock, AR

## Abstract

**Introduction:**

Teaching burn wound care can be challenging in the context of a busy clinic. Time and resource barriers make it difficult to provide structured and impactful education, leaving patients without crucial knowledge about wound care procedures. Effective teaching requires learners to be attentive, willing to learn, and for education to match their learning style. The Burn Clinic provides care to over 2200 outpatients annually. Historically, standard wound care (SWC) utilized hands-on demonstration and written instructions. Often, patients would request to record the wound care education on their personal devices. This elicited concerns for safety and privacy, with the risk of these videos being breeched or posted online. To improve education and safety, standardized wound care videos (WCV) were implemented. These WCV utilized a script written by the burn specialty nurse and reviewed by multidisciplinary members of the team. Thirteen individual WCV were created and made accessible to patients and caregivers via quick response (QR) code. This project assessed patient and caregiver confidence with wound care before and after implementation of WCV.

**Methods:**

Prior to the implementation of WCV, patients received SWC only. Following one to two weeks of home wound care, a cohort of clinic patients were given a survey. The survey assessed their confidence with home wound care and ability to identify the needed supplies based on the education received at the initial clinic visit. Additionally, learners were asked their preferred learning styles and methods used to teach wound care at their last clinic visit. A second cohort received the SWC in addition to WCV accessible by QR code. After one to two weeks of home wound care, the second cohort completed the same follow-up survey.

**Results:**

Greater than 40% of each cohort indicated one of their preferred learning styles was watching videos. In cohort 1 (n = 57), 93% reported being completely or somewhat confident with wound care learned by in person teaching and written instructions (SWC). With the addition of WCV, this increased to 96.3% in cohort 2. In cohort 1, 94.7% reported being completely or somewhat confident with identifying needed supplies, which increased to 100% for cohort 2. In cohort 2, 48.1% of respondents utilized the WCV.

**Conclusions:**

Providing WCV, in addition to the standard, hands-on teaching can improve confidence and competence with wound care. This improvement has the potential to impact wound healing and overall recovery from a burn injury.

**Applicability of Research to Practice**:: Engaging health professionals in creating WCV to bolster clinic teaching can provide a much needed resource for those performing wound care outside the hospital setting. WCV mitigate the privacy concerns created by recording on personal devices.

**Funding for the study:**

N/A.

